# Association of Open-Angle Glaucoma Loci With Incident Glaucoma in the Blue Mountains Eye Study

**DOI:** 10.1016/j.ajo.2014.09.020

**Published:** 2015-01

**Authors:** Kathryn P. Burdon, Paul Mitchell, Anne Lee, Paul R. Healey, Andrew J.R. White, Elena Rochtchina, Peter B.M. Thomas, Jie Jin Wang, Jamie E. Craig

**Affiliations:** a Department of Ophthalmology, Flinders University, Adelaide, Australia; b Menzies Research Institute Tasmania, University of Tasmania, Hobart, Australia; c Centre for Vision Research, Department of Ophthalmology and Westmead Millennium Institute, University of Sydney, Sydney, Australia; d Save Sight Institute, University of Sydney, Sydney, Australia; e Department of Ophthalmology, Ipswich Hospital, Ipswich, United Kingdom

## Abstract

**Purpose:**

To determine if open-angle glaucoma (OAG)-associated single nucleotide polymorphisms (SNPs) are associated with incident glaucoma and if such genetic information is useful in OAG risk prediction.

**Design:**

Case-control from within a population-based longitudinal study.

**Methods:**

study population: Individuals aged over 49 years of age living in the Blue Mountains region west of Sydney and enrolled in the Blue Mountains Eye Study. observation: Cases for this sub-study (n = 67) developed incident OAG between baseline and 10-year visits, in either eye, while controls (n = 1919) had no evidence for OAG at any visit. All participants had an ocular examination and DNA genotyped for reported OAG risk SNPs. main outcome measure: Incident OAG.

**Results:**

Two loci also known to be associated with cup-to-disc ratio as well as OAG (9p21 near *CDKN2B-AS1* and *SIX1/SIX6*) were both significantly associated with incident OAG in the Blue Mountains Eye Study cohort (*P* = .006 and *P* = .004, respectively). The *TMCO1* locus was nominally associated (*P* = .012), while the *CAV1/CAV2* and 8q22 loci were not associated. Multivariate logistic regression and neural network analysis both indicated that the genetic risk factors contributed positively to the predictive models incorporating traditional risk factors.

**Conclusions:**

This study shows that previously reported genetic variations related to OAG and cup-to-disc ratio are associated with the onset of OAG and thus may become useful in risk prediction algorithms designed to target early treatment to those most at risk of developing glaucoma.

Open-angle glaucoma (OAG) is one of the most common causes of blindness worldwide and the number of affected individuals is expected to increase as the population ages.^1^ It is characterized by the progressive loss of retinal ganglion cells, resulting in visual field defects beginning in the periphery and progressing centrally.

Current guidelines for the Screening, Prognosis, Diagnosis, Management, and Prevention of Glaucoma^2^ state that individuals at low risk of conversion from glaucoma suspect or ocular hypertension to glaucoma should be monitored, and those at high risk should be considered for treatment. The determination of who is at risk is based on a range of clinical risk factors, such as intraocular pressure, migraine, family history, and central corneal thickness.^2^ The genetic component of glaucoma risk is well recognized. Several high-penetrance genes have been described^3^, ^4^ and genetic testing is available for some of these.^5^ However, most patients do not carry mutations, and thus the contribution of genetics in risk prediction is currently limited to knowledge of family history, which is notoriously unreliable.^6^

Several common genetic variants increasing the risk of OAG have recently been identified through genome-wide association studies (GWAS; Table 1). Three studies of white individuals have collectively identified 5 loci.^7^, ^8^, ^9^ Loci reaching genome-wide significance levels include *TMCO*1 on chromosome 1q24,^7^ CAV1/CAV2^8^ on 7q31, a regulatory region on 8q22,^9^ the 9p21 locus near *CDKN2B-AS1*,^7^, ^9^ and *SIX1/SIX6*^9^ on 14q23. Several of these loci have also been associated with OAG-related quantitative traits, including intraocular pressure (IOP) and vertical cup-to-disc ratio (VCDR). However, reports from these cross-sectional studies did not distinguish whether the SNPs are associated with the initiation or progression of OAG. Different genetic factors may be involved with these 2 phases. Two of the loci (9p21 and *TMCO1*) have been identified in an advanced OAG cohort, suggesting they could be important in disease progression leading to the observed enrichment in advanced disease. Both regions are also associated with less severe OAG cases, indicating they may also be important to the vulnerability to OAG and its initiation.^7^

**Table 1:** Published Genome-Wide Significant Single Nucleotide Polymorphism Associations With Open-Angle Glaucoma and Associated Traits

Locus	SNP (Allele)	Gene	Phenotype	Country	Results	Reference
1q24	rs4656461(G)	*TMCO1*	Advanced POAG	Australia	*P* = 6.1 × 1^−10^ OR = 1.68	Burdon et al^7^
	rs755523(C)	*TMCO1*	IOP	Netherlands	*P* = 1.6 × 10^−8^ B = 0.28	Van Koolwijk et al^16^
7q31	rs4236601(A)	*CAV1/CAV2*	POAG	Iceland	*P* = 5.0 × 10^−10^ OR = 1.36	Thorleifsson et al^8^
8q22	rs1521774(G)	Gene desert	NTG	USA	*P* = 1.35 × 10^−9^ OR = 0.62	Wiggs et al^9^
9p21	rs4977756(A)	*CDKN2B-AS1*	Advanced POAG	Australia	*P* = 4.7 × 10^−9^ OR = 1.50	Burdon et al^7^
	rs1412829(T)	*CDKN2B-AS1*	POAG	Australia	*P* = 2.93 × 10^−10^ OR = 1.45	Burdon et al^7^
	rs1063192(G)	*CDKN2B*	POAG	USA	*P* = 2.30 × 20^−17^ OR = 1.43	Wiggs et al^9^
			VCDR	Netherlands	*P* = 1.96 × 10^−14^ B = −0.014	Ramdas et al^17^
14q22	rs10483727(A)	*SIX1/SIX6*	POAG	USA	*P* = 3.87 × 10^−11^ OR = 1.32	Wiggs et al^9^
			VCDR	Netherlands	*P* = 9.30 × 10^−11^ B = 0.012	Ramdas et al^17^

B = beta; IOP = intraocular pressure; NTG = normal tension glaucoma; OR =odds ratio; *P* = *P* value; POAG = primary open-angle glaucoma; SNP = single nucleotide polymorphism; VCDR = vertical cup-to-disc ratio.

There have been no previous reports seeking to examine genetic risk associated with the onset of OAG. To fill in this gap of knowledge, we have undertaken an analysis in an older Australian cohort from the Blue Mountains Eye Study (BMES), to determine whether genetic analysis could inform on the likelihood of an individual's being diagnosed with glaucoma in the future. The BMES is a well-known longitudinal population-based study of ophthalmic health and disease that includes baseline and 5-year and 10-year follow-up data. This population is considered to be stable, homogeneous, and representative of the Australian population, but older compared to the average for the state of New South Wales, where it was recruited.^10^

## Methods

The Blue Mountains Eye Study was approved by the Human Research Ethics Committee of the University of Sydney for investigation of the epidemiology and genetics of ocular disease. The BMES has been described previously.^10^ Briefly, the BMES is a population-based study of individuals living in the Blue Mountains region west of Sydney, Australia. Any permanent, noninstitutionalized resident of the defined geographic region born before January 1, 1943 (aged over 49 years at time of recruitment) and able to give written informed consent was eligible for enrollment in BMES and was contacted by door-to-door canvassing. Participants underwent a baseline visit, with follow-up at 5 years and at 10 years. At baseline, all participants received a detailed eye examination, including applanation tonometry, suprathreshold automated perimetry (Humphrey 76-point test, followed by 30-2 fields [Humphrey Visual Field Analyser 630 with StatPac 2, Humphrey Instruments, Inc, San Leandro, California, USA]), and stereoscopic optic disc photography (Carl Zeiss Australia, Sydney, New South Wales, Australia). The current sub-study consisted of a case-control design from within the BMES cohort study. Participants with normal threshold or suprathreshold field tests and no sign of glaucoma at the baseline visit were included in the current study. Participants with OAG at baseline (prevalent OAG) were excluded. As previously reported,^11^ incident OAG cases were defined as participants free of OAG at baseline who showed glaucomatous field loss on full-threshold perimetry (Humphrey 24-2 or 30-2), which matched the optic disc appearance, at either the 5-year or 10-year follow-up visit, without reference to intraocular pressure. Patients with pseudoexfoliation syndrome were not excluded (n = 7). DNA was extracted from peripheral whole blood using standard techniques. Genotyping was performed on the HumanHap670 array (Illumina, San Diego, California, USA) as part of the Wellcome Trust Case Control Cohort 2 Genome-Wide Association Study. Data were cleaned and genotypes called as previously described.^12^ No significant population stratification was detected in this population.^12^

Single nucleotide polymorphisms (SNPs) were selected for analysis if they had been previously reported to be associated with OAG (including normal tension glaucoma) at genome-wide significance in white populations. The reported SNPs with the smallest *P* values at each locus were chosen for this analysis. In the case of the 9p21 locus reported independently in 2 papers,^7^, ^9^ the top SNP from each paper was chosen, as well as a third SNP at genome-wide significance in the replication cohorts of Burdon and associates (rs1412829).^7^ We hypothesize that if this SNP had been typed in the discovery cohort for this study, it would likely have been the top-ranked SNP at this locus. Seven SNPs at 5 loci were chosen for analysis in total.

Power calculations were conducted with the Genetic Power Calculator.^13^ This study had 77% power to detect an association at a SNP with an allele frequency of 30% and an odds ratio of 1.6 under an additive model at a *P* value of .007, assuming a population disease prevalence of 5.67%.^14^ These parameters are similar to those reported for most of these loci in cross-sectional studies of OAG genetics.

Differences in the demographics of the available cohort were assessed using IBM SPSS Statistics V20. Association analysis was conducted under a univariate allelic model and also using logistic regression under an additive model adjusted for baseline measurements of age, sex, mean IOP of both eyes, mean cup-to-disc ratio of both eyes, mean disc diameter of both eyes, and systolic and diastolic blood pressure using Plink.^15^ Statistical significance was set to *P* < .007 under a Bonferroni correction, to account for the 7 SNPs tested. One associated SNP from each significant or nominally significant locus and the clinical variables were included in a logistic regression model using IBM SPSS Statistics V20. SNPs were coded to the number of OAG risk alleles carried by each participant at each SNP (0, 1, or 2). Collinearity between variables in the model were assessed by calculating the tolerance and the variance inflation factor (VIF). No collinearity was detected (no VIF >2).

The rank importance of each model component was also assessed using a large population of neural networks (produced using Matlab; The MathWorks, Inc, Natick, Massachusetts, USA). A neural network can be thought of as a small machine capable of learning. It is trained by exposure to a dataset comprising inputs (for example, the characteristics of horses in a race) and outputs (the winning horse). After each round of training, the link strengths within the network are changed, and further training is undertaken until its predictive performance on a previously unseen “validation” dataset no longer improves. The resulting network's performance is then measured using a final, also unseen “test” dataset. In this study, each neural network drew its inputs from unique subset of 7 SNPs and 7 clinical variables (age, sex, diastolic and systolic blood pressure, cup-to-disc ratio, IOP, and disc diameter). To cover all possible permutations of these 14 inputs, 16 383 neural networks were required.

Each neural network was trained and tested with a cohort comprising glaucoma patients (n = 67) and an equal number of randomly selected controls: 70% of the cohort was used to train the network, 15% to validate its performance during training, and the remaining 15% were unseen during training and were used to test the final performance of each network. Each neural network was trained and tested 20 times. In separate analyses, controls were either age matched to within 2 years of incident cases or not age matched. An error score was calculated to describe the performance of each neural network: the error score was the proportion of cases incorrectly classified (as glaucoma or as not glaucoma) by the network. Ranking of the importance of input variables (clinical parameters and SNPs) was achieved by ranking their influence on neural network error score. If the presence of a particular SNP or clinical variable (among the neural network's input variables) reduced the error score, that SNP or variable can be considered to make a positive contribution to the performance of the network (ie, it is of useful predictive value).

## Results

The BMES cohort consisted of 1986 individuals with follow-up phenotype data at either the 5-year, 10-year, or both visits with genotypes available (Table 2). Of the 1986 participants, there were 67 incident OAG cases over the full 10-year follow-up period. At baseline, the incident OAG cases were significantly older than controls (*P* < .001) and had a higher proportion of female subjects (*P* = .009). IOP and VCDR at the baseline visit were also significantly different between those who later developed OAG and those who did not (Table 2), as was systolic blood pressure. These features of this cohort have been previously reported.^11^

**Table 2:** Baseline Characteristics of the Eligible Blue Mountains Eye Study Participants by Glaucoma Status

	Incident OAG (Cases)	No OAG (Controls)	*P* Value
N	67	1919	
Sex (% female)	73%	57%	.009
Age	68.9 ± 7.9	63.8 ± 8.3	<.001
Mean IOP (mm Hg)	17.6 ± 2.8	15.9 ± 2.6	<.001
Mean VCDR	0.53 ± 0.11	0.42 ± 0.12	<.001
Mean DD (mm)	1.52 ± 0.18	1.51 ± 0.17	.570
Systolic BP (mm Hg)	151.6 ± 21.2	144.0 ± 20.4	.005
Diastolic BP (mm Hg)	85.7 ± 9.5	83.4 ± 9.6	.053

BP = blood pressure; DD = disc diameter; IOP = intraocular pressure; OAG = open-angle glaucoma; VCDR = vertical cup-to-disc ratio.

Data presented as mean ± standard deviation.

Association analysis indicates that incident OAG was associated with SNPs at 3 of the 5 loci tested (Table 3). Significant association under an allelic test was seen at rs1412892 (*P* = .006) at the 9p21 locus as well as rs10483727 (*P* = .004) at the *SIX1/SIX6* locus. Additional SNPs at 9p21 and also at *TMCO1* were nominally significant but did not survive after correction for multiple comparisons. The SNPs at the 8q22 and *CAV1/CAV2* loci did not show association with incident glaucoma. Adjustment for covariates under an additive genetic model showed association at the same SNPs, although only *SIX1/SIX6* remained significant after correction for testing 7 SNPs (*P* ≤ .007) (Table 3).

**Table 3:** Association of Genome-Wide Significant Open-Angle Glaucoma Loci With 10-Year Incident Glaucoma in the Blue Mountains Eye Study

Locus	CHR	SNP	Position (bp)^a^	A1/A2	Frequency A1		Univariate Analysis		Multivariate Analysis^b^	
					Cases	Controls	*P* Value	OR (95% CI)	*P* Value	OR (95% CI)
*TMCO1*	1	rs4656461	163953829	G/A	0.187	0.116	.013	1.74 (1.12–2.72)	.022	1.79 (1.09–2.95)
*CAV1/CAV2*	7	rs4236601	115949965	A/G	0.313	0.268	.239	1.25 (0.86–1.81)	.329	1.21 (0.82–1.78)
8q22	8	rs1521774	106048166	G/A	0.366	0.324	.314	1.20 (0.84–1.72)	.443	1.16 (0.80–1.67)
9p21	9	rs1063192	21993367	A/G	0.642	0.549	.033	1.47 (1.03–2.11)	.103	1.37 (0.94–2.01)
9p21	9	rs1412829	22033926	A/G	0.687	0.568	.006	1.67 (1.15–2.42)	.025	1.57 (1.06–2.32)
9p21	9	rs4977756	22058652	A/G	0.687	0.591	.027	1.52 (1.05–2.20)	.109	1.37 (0.93–2.02)
*SIX1/SIX6*	14	rs10483727	60142628	A/G	0.508	0.384	.004	1.66 (1.17–2.34)	.007	1.70 (1.16–2.50)

A1 = allele 1; A2 = allele 2; CHR = chromosome; CI = confidence interval; OR = odds ratio; SNP = single nucleotide polymorphism.

a SNP positions are given in genome assembly hg18 in base pairs (bp). All analyses are with respect to the reported open-angle glaucoma risk allele (A1).

b Multivariate analysis is adjusted for baseline characteristics age, sex, mean intraocular pressure, cup-to-disc ratio and disc diameter of both eyes, and systolic and diastolic blood pressure.

When all covariates and the 3 associated loci (*TMCO1*, 9p21, and *SIX1/SIX6*) were included in a single regression model, all variables except blood pressure contributed significantly to the model (Table 4).

**Table 4:** Logistic Regression Model Showing Association of Each Clinical Variable and Associated Polymorphism With 10-Year Incident Open-Angle Glaucoma in the Blue Mountains Eye Study

Variable	B	SE	*P* Value	OR (95% CI)
Female sex	0.930	0.305	.002	2.53 (1.39–4.61)
Age (y)	0.064	0.017	<.001	1.07 (1.03–1.10)
Mean IOP (mm Hg)	0.217	0.048	<.001	1.24 (1.13–1.36)
Mean VCDR	0.827	0.135	<.001	2.29 (1.76–2.98)
Mean DD (mm)	−1.803	0.838	.031	0.17 (0.03–0.85)
Systolic BP (mm Hg)	−0.005	0.008	.559	1.00 (0.98–1.01)
Diastolic BP (mm Hg)	0.021	0.018	.245	1.02 (0.99–1.06)
rs4656461 (no. of risk alleles)	0.608	0.258	.018	1.84 (1.11–3.04)
rs1412829 (no. of risk alleles)	0.439	0.205	.032	1.55 (1.04–2.32)
rs10483727 (no. of risk alleles)	0.525	0.200	.009	1.69 (1.14–2.50)
Constant	−16.221	2.348		

B = maximum likelihood estimate; BP = blood pressure; CI = confidence interval; DD = disc diameter; IOP = intraocular pressure; OR = odds ratio; SE = standard error of B; VCDR = vertical cup-to-disc ratio.

The population of neural networks was used to compare the rank importance of variables in the predictive model both with and without age matching between controls and incident cases (Table 5). As expected, when not age matched, vertical cup-to-disc ratio, age, and intraocular pressure rank the highest for predicting incident OAG. The top-ranked SNP in this analysis is at the *SIX1/SIX6* locus, which also showed the strongest genetic association. When cases and controls were closely age matched the rank order of variables changed, likely indicating an interaction between age and the other variable, although vertical cup-to-disc ratio and intraocular pressure are still the most predictive variables. In this situation the SNP at the *TMCO1* locus was most predictive. Of note, in both analyses, all SNPs significantly associated with incident OAG under the traditional statistics contribute positively to the neural network and improve its ability to predict incident OAG.

**Table 5:** Neural Network Ranking and Contribution to Risk Prediction of Variables Associated With 10-Year Incident Open-Angle Glaucoma Risk in the Blue Mountains Eye Study

Rank	Age Matched		Not Age Matched	
	Variable	Contribution^a^	Variable	Contribution^a^
1	Mean VCDR	0.034777	Mean VCDR	0.041231
2	Mean IOP	0.030919	Age	0.031223
3	Female sex	0.008209	Mean IOP	0.028952
4	rs4656461	0.007138	rs10483727	0.004189
5	Diastolic BP	0.004934	Diastolic BP	0.004119
6	rs10483727	0.001923	Systolic BP	0.004102
7	rs4236601	0.001604	rs4656461	0.003295
8	rs1412829	0.000489	rs1412829	0.001517
9	Systolic BP	−0.00073	Mean DD	−0.00070
10	Mean DD	−0.00084	rs4236601	−0.00224
11	rs4977756	−0.00228	Sex	−0.00249
12	rs1063192	−0.00481	rs1063192	−0.00361
13	rs1521774	−0.00552	rs4977756	−0.00408
14	Age	−0.00748	rs1521774	−0.00922

BP = blood pressure; DD = disc diameter; IOP = intraocular pressure; VCDR = vertical cup-to-disc ratio.

a The contribution of each variable is defined as the reduction in neural network predictive error it confers.

## Discussion

The selected loci have been discovered through genome-wide association studies for OAG (*SIX1/SIX6, CAV1/CAV2*),^8^, ^9^ advanced OAG (9p21, *TMCO1*),^7^ or normal tension glaucoma (9p21, 8q22).^9^ In addition, variation at *TMCO1* has been associated with intraocular pressure,^16^ while 9p21 and *SIX1/SIX6* are associated with cup-to-disc ratio^17^ in normal individuals. We provide evidence for association at *SIX1/SIX6,* 9p21, and nominally at *TMCO1* with incident OAG. Thus, loci associated with advanced glaucoma and relevant biometric traits are also associated with the initial onset of OAG (incidence). Those SNPs discovered in previous cohorts with typical (nonadvanced) OAG are not found to be associated with OAG incidence in our cohort, although power to detect weaker associations or those at rarer SNPs is limited. The association of sex with incident OAG in the cohort has been previously reported,^11^ as has the higher-than-expected level of hypertension in the BMES cohort.^18^, ^19^

The current cohort was sufficiently powered to detect an odds ratio of ∼1.6. This is larger than those observed in the original discovery cohorts of cross-sectional (prevalent OAG) patient recruitment, although significant effects were still observed in this study, suggesting that the SNPs may be more important in predicting disease onset than progression, or that the true effect size is larger than previously reported. However, larger prospective cohorts will be needed to properly assess the 8q22 and *CAV1/CAV2* loci in particular.

A nominal association was observed at *TMCO1*. This SNP has a lower allele frequency than others in the study (11% in controls) and the finding did not reach significance here in the context of multiple testing, owing to the lower power of this study (∼36%) to detect an effect at the minor allele frequency of 11%. We have previously reported an association of this locus with prevalent OAG in the BMES cohort with odds ratio (OR) = 1.57, *P* = .022.^7^ The odds ratio for incident OAG reported in the current study was larger (OR = 1.74, *P* = .013) despite the smaller sample size. We thus conclude that *TMCO1* is also confirmed to be associated with incident OAG.

The current study shows that OAG loci that are associated with OAG-relevant ocular parameters (cup-to-disc ratio and intraocular pressure) are specifically associated with OAG incidence independently of other known risk factors. This suggests that these loci are responsible at least in part for the initiation of OAG, consistent with their role in determination of these risk factor traits, which are themselves predictive for OAG development. We show also that the loci specifically associated with advanced glaucoma may also be important in initiation of OAG, and thus could be important in risk stratification among glaucoma suspect and early glaucoma patients.

The multivariate logistic regression model indicates that mean vertical cup-to-disc ratio at baseline is by far the most significant predictor of incident glaucoma in this cohort, while in the context of other parameters blood pressure contributes almost nothing to this model. Although vertical cup-to-disc ratio is a well-recognized parameter in the prediction of OAG risk, the accuracy of prediction based solely on this parameter is poor owing to disc appearance in preclinical and early glaucomatous damage overlapping with the normal range of this trait. Predictive accuracy for the individual patient should be improved by the inclusion of other variables, including genetics. With the genetics tools available at this time, discriminatory power above and beyond that achievable with clinical risk factors is minimal; however, ongoing research uncovering the genetic basis of OAG is likely to lead to better risk prediction models.

Neural networks allow an alternative approach to estimating the usefulness of clinical and genetic variables in predicting incident glaucoma. Input variables that are predictive of incident glaucoma naturally benefit the performance of the network. However, we see that those variables of trivial or no predictive value negatively affect the performance of the network: their inclusion necessarily makes the network structure more complex, which will lead to increased noise in the network. Neural networks are therefore helpful in distinguishing those patient characteristics that might help the clinician to predict glaucoma incidence and those that will merely overload him or her with unhelpful information. This approach could easily be expanded to larger datasets where specific combinations of variables that are particularly beneficial might become apparent. The matching of age (an important OAG risk factor) between cases and controls in the neural network analysis resulted in the *TMCO1* SNP, rs4656461, becoming the highest-ranked genetic variable. This is consistent with a previously reported finding of the association of this SNP with age of onset of OAG.^20^

Each of the associated SNPs in the logistic regression model also contributed positively in the neural network. Thus, the combination of IOP, disc parameters, and genotype at-risk SNPs could improve the accuracy of OAG risk prediction, which in turn will inform early treatment decisions for those most likely to develop this blinding disease.
